# Soybean Foliar Deposition and Airflow Distribution Interrelated to Nozzle Type and Boom Travel Direction in Wind Tunnel

**DOI:** 10.3390/plants15071032

**Published:** 2026-03-27

**Authors:** João Paulo Arantes Rodrigues da Cunha, Rone Batista de Oliveira, Gabriel de Souza Lemes, Erdal Ozkan, Hongyoung Jeon, Heping Zhu

**Affiliations:** 1 Institute of Agrarian Sciences, Federal University of Uberlândia, Uberlândia 38408-100, Brazil; 2 Department of Food, Agricultural and Biological Engineering, The Ohio State University, Wooster, OH 44691, USA; rone@uenp.edu.br (R.B.d.O.); desouzalemes.1@osu.edu (G.d.S.L.); 3 Center of Agrarian Sciences, Northern Paraná State University, Bandeirantes 86400-000, Brazil; 4 Department of Food, Agricultural and Biological Engineering, The Ohio State University, Columbus, OH 43210, USA; ozkan.2@osu.edu; 5 Application Technology Research Unit, United States Department of Agriculture, Wooster, OH 44691, USA; hongyoung.jeon@usda.gov (H.J.); heping.zhu@usda.gov (H.Z.)

**Keywords:** canopy penetration, droplet size, pesticide application technology, wind effect

## Abstract

Spray deposition and coverage within soybean canopies remain critical challenges for achieving effective pesticide applications, particularly under windy conditions. This research investigated the influence of wind speed, boom travel direction relative to wind direction, and nozzle type on droplet deposition, coverage uniformity, canopy penetration, and airflow distributions inside soybean canopies under controlled wind-tunnel airflow. Spray deposition, analyzed using a fluorometric tracer, and coverage, quantified with water-sensitive papers, were assessed in R3-stage soybeans in an 18-m wind tunnel using XR (perpendicular spray) and 3D (38° angle) flat fan nozzles under varying air speeds and boom travel directions in the wind tunnel. Potted plants were placed in the wind tunnel to mimic soybeans grown in field conditions. Droplet sizes of the nozzles were measured using a laser imaging particle sizing system. Airflow velocity and turbulence within the soybean canopy were investigated with a 3-D hot-film anemometer system. The results indicated that wind and boom direction were the main influential factors for spray coverage and deposition. The top canopy position, exposed to the highest air-turbulence intensity, received the greatest deposition, whereas the middle and bottom positions, characterized by lower turbulence, exhibited sharp declines in both deposition and coverage regardless of treatment. The 3D nozzle provided greater coverage and deposition than the XR nozzle only under no-wind conditions; however, under wind conditions, equivalent performance was observed from both nozzles. Therefore, it was essential to incorporate wind conditions and canopy structures into consideration when choosing nozzles to maximize spray penetration and achieve efficient and effective spray applications for soybeans.

## 1. Introduction

Field spray experiments are often impacted by numerous uncontrollable variables which hinder a clear understanding of wind effects on droplet penetration into crop canopies under varying operational conditions. Wind-tunnel studies, therefore, offer a controlled and repeatable environment, enabling a precise evaluation of airflow-droplet-canopy interactions [1,2].

Crop and weed foliage form complex three-dimensional structures that intercept, deflect, and retain spray droplets, thus reducing penetration as canopy density increases [3]. Although advancements in spray technology have improved understanding of droplet size effects and drift reduction, their influence on canopy penetration and foliar coverage remains inconsistent and often unpredictable [2]. Optimizing application techniques is therefore essential to ensure adequate droplet delivery to dense canopies such as soybean (*Glycine max* (L.) Merr.), where the lower canopy layers are particularly difficult for droplets to reach [4].

Spray coverage in the middle and lower canopy layers is greatly reduced by canopy density and structure because they impede droplet penetration along the vertical canopy profile [5]. Investigations consistently report reduced deposition in the middle and lower canopy, regardless of nozzle type [5,6]. In recent years, spray nozzles with various designs such as multiple orifices to alter spray trajectories and droplet size classes have become available, providing applicators more options to achieve various application goals [7]. Among these technologies, angled flat-fan nozzles have been developed to enhance deposition in the lower canopy [8].

The effectiveness of angled nozzles in improving canopy penetration, however, varies across crops. A discharging angle of such nozzles is especially relevant in wheat (*Triticum aestivum* L.), where twin-pattern nozzles increase coverage for head blight (*Fusarium graminearum*) control. However, the same nozzles appear less suited for soybean diseases, which require spray deposition on middle and lower canopy surfaces [9]. Spray penetration is primarily influenced by the droplet size spectrum, which is mainly governed by nozzle type, spray mixture, and nominal flow rate, whereas spray angle plays a relatively minor role [8].

Most previous studies on spray penetration were conducted either in the field using plants under varied wind conditions or in wind tunnels using artificial plants. However, varied wind conditions during the spray penetration experiments conducted in field settings may influence the movement of spray droplets leading to different results between studies. In addition, artificial plants do not represent the complexity of real plants in leaf overlap, orientation, and spatial arrangement. These concerns highlight the importance of conducting experiments that combine realistic canopy structures with controlled airflow. More recently, Castilho Theodoro et al. [10] evaluated four nozzle types in a wind tunnel and reported that nozzle selection can influence spray coverage in soybeans. However, regardless of nozzle types, the upper canopy consistently received the highest coverage, followed by the middle and lower layers of the canopy, reinforcing the persistent challenge of achieving adequate deposition in lower canopy zones.

Additionally, most wind-tunnel studies examined only one travel direction of the spray boom under laminar airflow, overlooking the interaction between boom movement and wind direction. This relative-motion effect may alter turbulence and droplet trajectories to influence vertical penetration in the canopy. Consequently, the influences of boom travel direction and nozzle orientation on spray deposition are unclear, although similar work has been conducted [2,10]. Thus, the objectives of this research were to determine the influence of nozzle type and boom travel direction on spray coverage and deposition and to investigate airflow distributions inside soybean canopies under controlled wind-tunnel airflow.

## 2. Results and Discussion

### 2.1. Droplet Size

Table 1 presents the volumetric droplet diameters, V_153_, and relative span index for sprays produced by XR 11003 and 3D 10003 nozzles at a 30% duty cycle and 276 kPa pressure. The results indicate that the 3D 10003 nozzle generated a larger droplet size spectrum than the XR 11003. In particular, the D_v50_ for the 3D 10003 nozzle was 248 µm, representing a 50.7% increase compared to 164 µm for the XR 11003 nozzle. This shift towards larger droplet sizes was consistent with the higher D_v10_ and D_v90_ values observed for the 3D nozzle. The differences in droplet size distribution were reflected in the V_153_ values, with the XR 11003 producing a higher proportion of fine droplets (44.8%) than the 3D 10003 nozzle (23.0%).

These differences might have some influence on spray deposition and drift potential. For example, droplets from XR 11003 tended to be slightly smaller, which could improve spray coverage and deposition uniformity across the canopy, but could also increase drift susceptibility due to lower mass and longer suspension time. In contrast, the 3D 10003 produced relatively larger droplets, potentially reducing drift risk, but having a potential reduction in spray coverage. The similar relative span index values for the two nozzles indicated comparable variability in droplet size distribution, suggesting that the practical differences between them were not significantly different (Table 1).

### 2.2. Air Velocity and Turbulence

**Figure 1:**
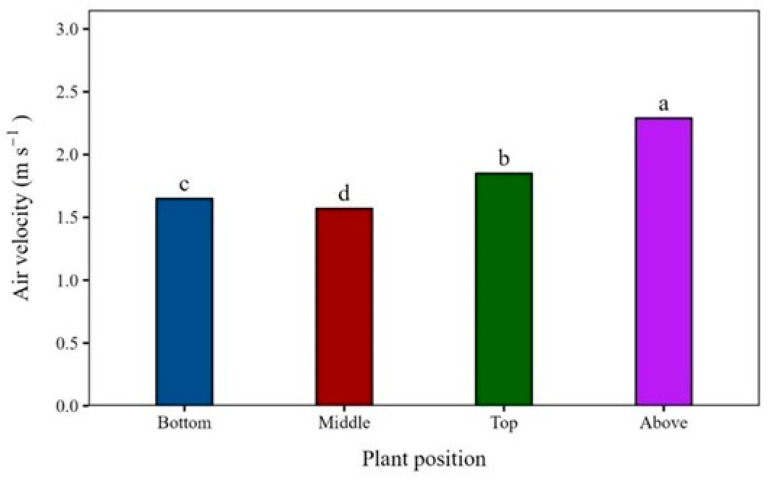
Mean resultant air velocity (m s^−1^) at different positions within the soybean canopy under a wind speed of 4.4 m s^−1^. Bars followed by different letters differ significantly according to Tukey’s test (*p* < 0.05). Experimental CV = 4.6%.

The mean resultant air velocity profile within the canopy (Figure 1) revealed a vertical gradient of air velocity resulting from the aerodynamic resistance imposed by the soybean foliage. Above the canopy (0.2 m), the mean air velocity reached approximately 2.3 m s^−1^, corresponding to about 50% of the wind speed (4.4 m s^−1^) in the tunnel without soybean plants. Within the foliage layer, airflow decreased slightly, with mean values of approximately 1.9 m s^−1^ at the upper canopy, 1.6 m s^−1^ at the mid-canopy, and 1.7 m s^−1^ at the lower canopy positions. This attenuation pattern reflected the drag forces and momentum absorption described by Baldocchi et al. [12], who reported a similar velocity distribution for soybean canopies.

The observed profile was consistent with prior research results on air movement within plant canopies, which described and characterized the vegetation as a porous medium that extracted momentum from the flow and dissipated kinetic energy through form drag and wake production [13]. As the air penetrated the canopy, its velocity decreased due to increased airflow resistance and small eddies around leaves and stems. The upper canopy acted as a transitional layer, where strong velocity gradients and enhanced turbulence typically occurred, while the airflow above the canopy gradually approached a logarithmic boundary-layer profile [14].

Reduced airflow within the canopy, combined with lower turbulence, might limit the transport of spray droplets by airflow. This might lead to uneven deposition patterns inside the canopy, as lower air velocities hindered droplet penetration and promoted accumulation on the upper leaves. Even under moderate-to-high external wind speeds, the combined effects of canopy porosity and leaf density substantially restricted internal ventilation, underscoring their decisive influence on spray application efficiency.

**Figure 2:**
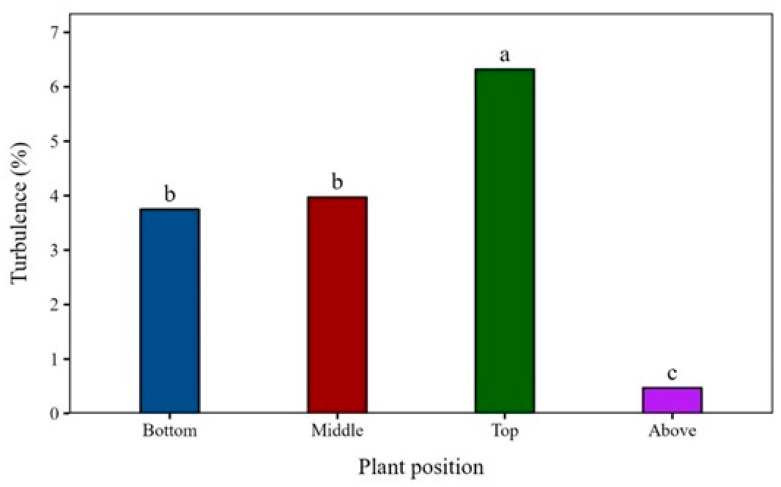
Turbulence intensity (%) at different positions within the soybean canopy under a wind speed of 4.4 m s^−1^. Bars followed by different letters differ significantly according to Tukey’s test (*p* < 0.05). Experimental CV = 16.4%.

The air turbulence above the soybean canopy stayed below 0.5% at a wind speed of 4.4 m s^−1^, indicating a region of low turbulence (Figure 2). Inside the canopy, however, the interaction of the airflow with leaves and branches substantially increased turbulence intensity, reaching 6.3% at the top of the plants. This zone corresponded to the transition between free flow and the obstructed canopy layer, where turbulent kinetic energy dissipation was greatest [15].

At the middle and bottom canopy positions, turbulence intensity was similar (~4%), reflecting the attenuation of airflow by the foliage and the energy absorption occurring in upper layers [12]. Such a reduction tended to limit droplet penetration during pesticide spraying, especially when combined with coarser droplet spectrum. The weakened air turbulence limited droplet dispersion and reduced downward momentum [16], which restricted spray penetration and favored droplet interception by the upper canopy strata.

These results demonstrated that even under moderate-to-high wind speeds (4.4 m s^−1^), the flow regime within the canopy remained highly heterogeneous and dependent on plant structures and leaf area index [12]. Enhanced turbulence near the canopy top promoted air and fine-droplet mixing due to the layer transition, whereas the turbulence attenuation in the lower layers reduced deposition and increased spatial variability, key determinants of spray penetration efficiency [7].

### 2.3. Spray Coverage

Analysis of variance indicated significant differences in spray coverage among treatments for the top (F = 15.26, *p* < 0.01), middle (F = 7.03, *p* < 0.01), and bottom (F = 7.51, *p* < 0.01) canopy positions, with distinct patterns observed at each level. The experimental coefficients of variation (CV) were 30.8%, 64.3%, and 60.3% for the top, middle, and bottom positions, respectively. The higher variabilities in the middle and bottom regions were expected due to the complex interactions among airflow, droplets, and canopies. In contrast, the top canopy exhibited more stable and predictable behavior.

**Figure 3:**
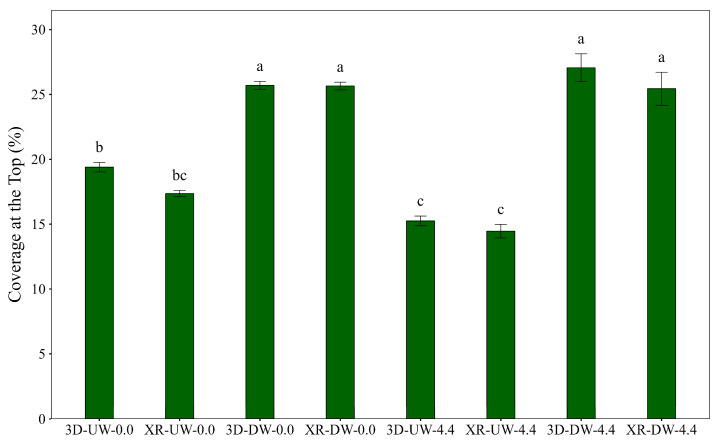
Coverage at the top of the soybean canopy as influenced by nozzle type, spray boom direction, and air speed. Bars followed by different letters differ significantly according to Tukey’s test (*p* < 0.05). Error bars represent the standard error of the mean. Experimental CV = 30.8%.

The combination of nozzle type, boom travel direction, and air speed significantly affected spray coverage at the top canopy position (Figure 3). Greater coverage was generally observed under DW conditions compared with UW conditions. However, this difference may also be influenced by the relative exposure of collectors within the wind tunnel under airflow conditions, particularly at the top canopy position. No consistent deposition gradient along the wind tunnel length was observed for the middle and lower canopy positions, which were the primary focus of this study. Under wind conditions and in the UW direction (3D-UW-4.4; XR-UW-4.4), coverage at the top canopy position was markedly reduced, suggesting that this configuration may be more sensitive to airflow disturbances.

The greater coverage observed at the top canopy layer seemed to be strongly linked to the full exposure of leaves in this level, without physical barriers limiting interception. However, the turbulence and air speed data (Figure 1 and Figure 2) indicate that these factors might also influence droplet transport and redistribution. Therefore, increased turbulence and air velocity at the top of the canopy might contribute to this pattern, influencing the effects of travel direction and wind speed on spray coverage. However, this interpretation should be considered as a hypothesis, because this aerodynamic perspective had only been lightly explored in the literature.

The 3D nozzle (38° angle) and the XR nozzle (perpendicular spray) showed similar coverage in the top position under DW conditions. Conversely, in the UW direction without wind, the 3D nozzle (3D-UW-0.0) produced higher coverage than the windy condition (3D-UW-4.4), a variation not observed for the XR nozzle (XR-UW-0.0 and XR-UW-4.4).

Cunha and Pereira [17] found that nozzle type and spray volume can produce significant differences in coverage at the top position, emphasizing that the combined effects of spray characteristics and airflow may lead to varying outcomes depending on the experimental context. However, their study was conducted under field conditions, where limited control of experimental variables was possible. Additionally, Foqué et al. [18] observed high experimental variability in top-canopy coverage, which often made it difficult to detect noticeable differences between treatments.

**Figure 4:**
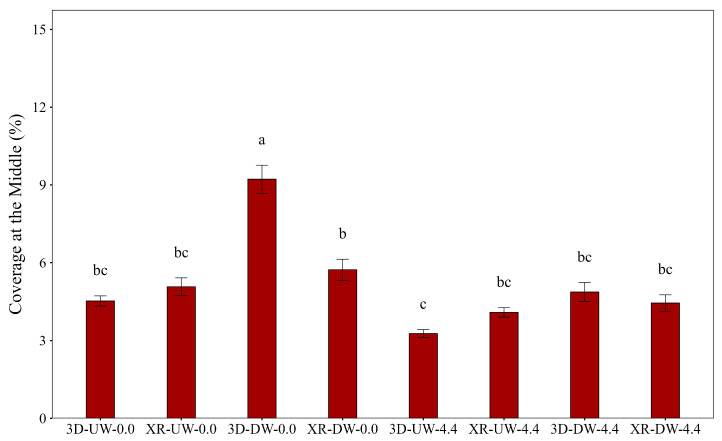
Coverage at the middle of the soybean canopy as influenced by nozzle type, spray boom direction, and air speed. Bars followed by different letters differ significantly according to Tukey’s test (*p* < 0.05). Error bars represent the standard error of the mean. Experimental CV = 64.3%.

In the middle position, the 3D-DW-0.0 treatment yielded significantly higher coverage (*p* < 0.05) than the other treatments (Figure 4). Conversely, under windy conditions combined with the UW travel direction (3D-UW-4.4), the 3D nozzle exhibited the lowest coverage, although the differences with other treatments were not significant.

While the 3D nozzle in the DW direction provided superior coverage in the middle part of soybean plants with no wind, regional climatic data indicate that such conditions are rare in the field. Silva et al. [19] identified wind as the primary meteorological constraint for pesticide application. Therefore, nozzle selection should focus on performance under windy conditions to match real-world situations.

**Figure 5:**
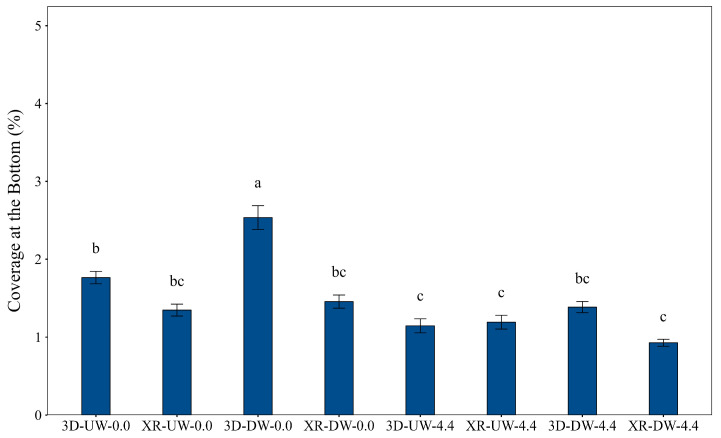
Coverage at the bottom of the soybean canopy as influenced by nozzle type, spray boom travel direction, and air speed. Bars followed by different letters differ significantly according to Tukey’s test (*p* < 0.05). Error bars represent the standard error of the mean. Experimental CV = 60.3%.

Figure 5 presents coverage in the bottom region, where treatment 3D-DW-0.0 again produced the highest value. However, the presence of wind caused significant reductions: coverage decreased by 45% in the DW direction (3D-DW-4.4) and by 35% in the UW direction (comparing 3D-UW-0.0 to 3D-UW-4.4). These results demonstrate the consistent negative impact of wind on 3D nozzle performance, regardless of boom travel direction. From a practical perspective, since boom direction varied naturally during field operations, spraying in windless or low wind conditions remained the most effective way to maximize coverage. Moreover, although the 3D nozzle outperformed the XR nozzle without wind, their performance was similar under windy conditions, confirming that wind was the dominant factor affecting coverage.

It should also be considered that the spray angle of the 3D nozzle (38°) may influence droplet trajectories under wind conditions. Although the 3D nozzle produced larger droplets than the XR nozzle, the inclined spray jets may increase droplet displacement when interacting with airflow. Therefore, the larger droplet size may not be sufficient to fully compensate for the angled spray trajectory. Further studies using similar nozzle designs with larger droplet spectra could help clarify the relative contributions of spray angle and droplet size to spray deposition under wind. These results suggest that spray orientation may play a critical role in nozzle performance under wind conditions.

### 2.4. Deposition

Consistent with the spray coverage results, analysis of variance indicated significant differences in deposition among treatments for the top (F = 2.62, *p* < 0.01), middle (F = 9.80, *p* < 0.01), and bottom (F = 10.71, *p* < 0.01) canopy positions, each with distinct distribution patterns. The experimental coefficients of variation were 15.8%, 44.7%, and 65.4% for the top, middle, and bottom positions, respectively. These values indicate that deposition uniformity within the canopy surpassed that of coverage, while also demonstrating a progressive increase in variability from the top to the bottom of the plants.

**Figure 6:**
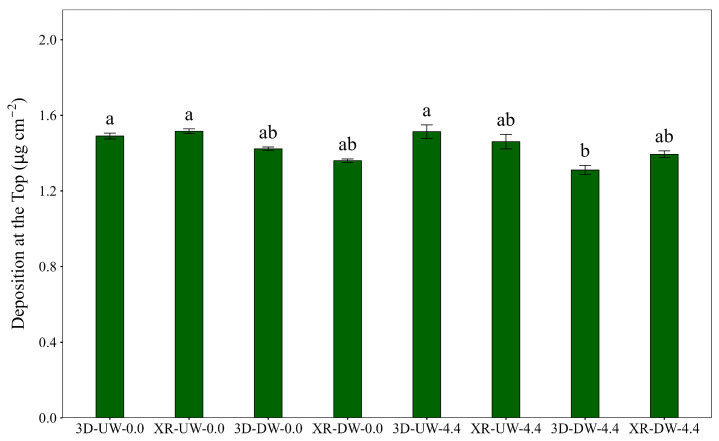
Deposition at the top of the soybean canopy as influenced by nozzle type, spray boom direction, and air speed. Bars followed by different letters differ significantly according to Tukey’s test (*p* < 0.05). Error bars represent the standard error of the mean. Experimental CV = 15.8%.

Figure 6 shows the deposition at the top position of the plants. A significant difference was observed between treatments 3D-UW-0.0, 3D-UW-4.4, and XR-UW-0.0 when compared with treatment 3D-DW-4.4. This indicates that under windy conditions with the boom traveling in the DW direction, spray deposition from the 3D nozzle could be lower than that of XR nozzles, although the differences might be incidental. The remaining treatments did not show significant differences. In this position, greater similarity among treatments was observed, as along with higher deposition than the middle and bottom positions.

It should be noted that water-sensitive paper coverage reflects droplet distribution on the target surface, whereas tracer quantification represents the total mass of spray deposited. Therefore, the two metrics do not necessarily show identical trends.

**Figure 7:**
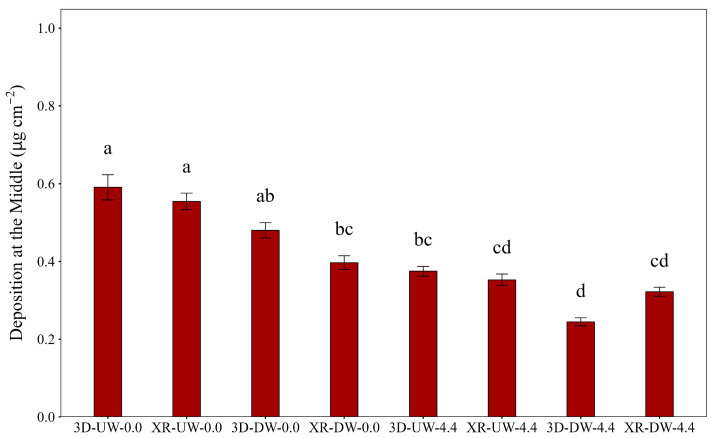
Deposition at the middle of the soybean canopy as influenced by nozzle type, spray boom direction, and air speed. Bars followed by different letters differ significantly according to Tukey’s test (*p* < 0.05). Error bars represent the standard error of the mean. Experimental CV = 44.7%.

The deposition at the middle canopy is presented in Figure 7. For both nozzle types, when the boom was moving in the UW direction, the presence of wind caused a significant reduction in deposition compared to no wind. This decrease was similar between spray nozzles, with averages of 36.6% for the 3D and 35.2% for the XR. Treatments 3D-UW-0.0 and XR-UW-0.0 showed the highest deposition, yet they did not differ from the 3D-DW-0.0 treatment.

In the middle section of the plants, the reduction in deposition was significant and became even more noticeable under wind conditions when using the 3D nozzle in the DW boom travel direction. The 3D nozzle produces spray jets oriented forward and backward relative to the travel direction, which may interact differently with airflow compared with conventional flat-fan nozzles. When the forward-oriented jet aligns with the wind direction, the horizontal component of droplet velocity may increase, potentially enhancing droplet displacement. However, the interaction between spray orientation, airflow, and boom movement creates a complex aerodynamic condition that may alter droplet trajectories before they reach the canopy, which could partially explain the lower deposition observed in the middle layer.

**Figure 8:**
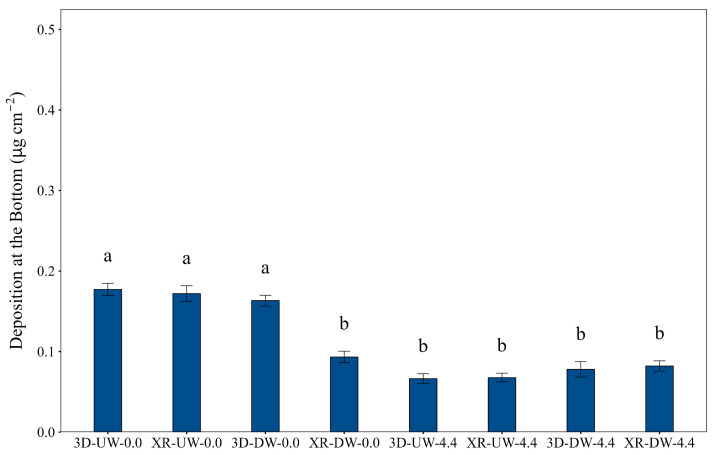
Deposition at the bottom of the soybean canopy as influenced by nozzle type, spray boom direction, and air speed. Bars followed by different letters differ significantly according to Tukey’s test (*p* < 0.05). Error bars represent the standard error of the mean. Experimental CV = 65.4%.

With the exception of the XR-DW-0.0 treatment, the treatments without wind showed significantly higher deposition (*p* < 0.05) in the bottom region of plants than the treatments under the wind condition (Figure 8).

In the bottom canopy, treatments without wind provided significantly higher deposition than those under windy conditions, aligning with the droplet fundamentals described by Cunha et al. [20]. Without wind, fine droplets travelled through the canopy by the effect of gravity, enabling some differentiation in nozzle performance and spray boom direction. However, the wind (4.4 m s^−1^) effectively dictated the movement of fine droplets to drift away instead of being deposited at this position.

A reduction in coverage and deposition from the top to the middle and bottom positions was observed across all treatments, albeit with varying magnitudes. As noted by Womac et al. [7], this indicates that droplet delivery to the bottom canopy remains a challenge that requires improved application technology. However, spray applications conducted in the absence of wind resulted in significant increases in both coverage and deposition in the middle and bottom sections of soybean plants.

The 3D flat-fan nozzle was developed based on the premise that it could improve spray coverage and deposition in the bottom canopy [21,22]. In the absence of wind, the 3D nozzle outperformed the XR nozzle in both deposition and coverage, but this advantage was not sustained under wind conditions even though the 3D nozzle produced 50.7% greater D_v50_ than the XR nozzle. Both XR and 3D nozzles exhibited acceptable coefficients of variation (below 7%) for volumetric distribution, indicating spray uniformity over a flat area [8], but this metric might not capture differences in effective coverage and deposition on the target, particularly within a complex three-dimensional canopy structure.

Changing the boom travel direction under windy conditions might influence spray interception of the plant canopy more than droplet size, thus overshadowing the design differences between the 3D and XR nozzles. Wind speed emerged as the dominant factor affecting deposition, more than nozzle types, particularly in the lower layers of the canopy, where aerodynamic effects outweighed nozzle design. These findings indicated that, although nozzle selection was still important, wind intensity should be the primary consideration when choosing the application technology and defining operational spray windows.

In addition to these effects, airflow dynamics inside the canopy might help explain the spatial patterns observed in deposition and coverage. In the upper canopy, high turbulence and air velocity were associated with increased coverage and deposition, whereas the middle and lower canopy showed simultaneous reductions in turbulence, air velocity, coverage, and deposition. However, although the decrease in turbulence and wind speed along the canopy coincided with the reductions in deposition and coverage, this similarity did not imply a direct cause-and-effect relationship. LAI and leaf overlap increased from the base of the canopy to the top, heavily influencing spray penetration and making it hard to separate the influences of airflow and canopy structure [17].

One important consideration was that reaching the lower canopy of soybean plants was inherently difficult, even in the absence of wind. When the upper leaves intercepted a large portion of the spray volume, little or no spray remained to penetrate the interior and lower parts of the canopy. This limitation was not related to the nozzle type used, but instead to the physical structure of the crop itself. In this context, increasing the total spray volume could help overcome this limitation, potentially allowing droplets to reach deeper into the canopy and improve overall deposition. However, increasing spray volume would also increase the spray application cost and potential environment contamination risk.

**Figure 9:**
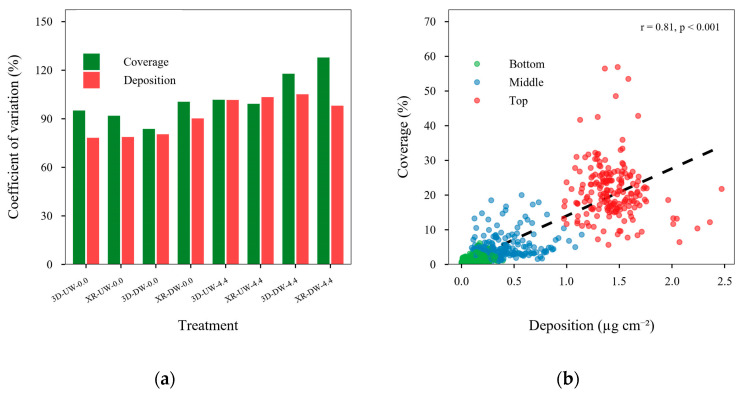
(**a**) Coefficient of variation in coverage and deposition across the three plant positions; (**b**) Pearson’s correlation between coverage and deposition.

Figure 9 presents the CV of coverage and deposition across the three plant positions for each treatment (Figure 9a), as well as the correlation between deposition and coverage (Figure 9b). It was observed that XR-DW-4.4 and 3D-DW-4.4 treatments increased the CVs for coverage and showed a greater difference in CV between deposition and coverage. These treatments resulted from the combined effects of DW direction and wind presence. The lowest CV values were found in treatments without wind, indicating a less variation application within the plant canopy.

Deposition showed positive correlation with coverage (r = 0.81, *p* < 0.001), indicating that higher deposition values were associated with greater coverage, regardless of canopy position (bottom, middle, and top). As shown in the scatter-plot, the top canopy (red points) received the highest deposition and coverage values, while the middle (blue points) and bottom (green points) layers generally had lower values. The dashed line represents the overall positive trend between deposition and coverage across all canopy positions, emphasizing that increased deposition consistently enhances coverage even in less exposed parts of the canopy.

## 3. Materials and Methods

### 3.1. Wind Tunnel

**Figure 10:**
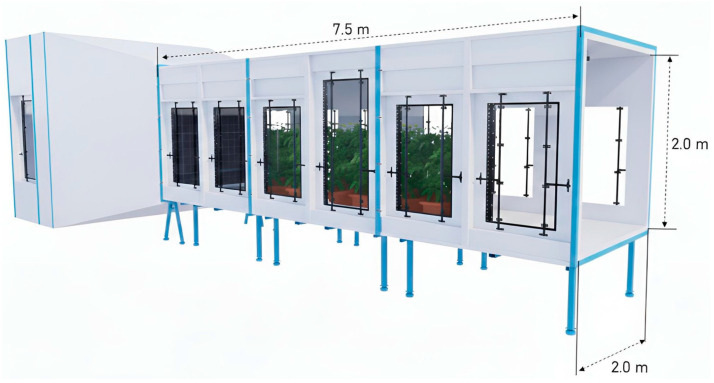
Wind tunnel used in the experiment.

An 18-m-long open-circuit wind tunnel, described in detail by Zhu et al. [23], located at The Ohio State University’s Wooster Campus (Wooster, OH, USA), was used to evaluate spray deposition in soybean promoted by different nozzle types under various wind conditions. The tunnel featured a test section measuring approximately 2 × 2 × 7.5 m and included a 75 kW centrifugal fan, a pre-chamber, a mixing chamber, a honeycomb flow straightener, and a variable-speed control system (Figure 10). The wind tunnel produced constant air velocities ranging from 0 to 10 m s^−1^ with turbulence intensities below 5% [23].

The wind tunnel also included a moving spray boom equipped with three nozzle bodies spaced 0.5 m apart, installed 0.6 m downstream from the honeycomb. The boom was mounted on a vertical post attached to a 6.0 m-long track positioned on the ceiling of the wind tunnel test section. The boom was moving at a constant speed of 0.9 m s^−1^, with its height maintained at 0.5 m above the top of the soybean canopy during all tests.

### 3.2. Soybean Plants

Soybean plants (*Glycine max* [L.] Merr., variety 36320236 Xitavo^®^) were cultivated in 0.68 m long, 0.30 m wide and 0.24 m deep rectangular plastic pots. Each pot contained eight plants arranged in a single central row, corresponding to a field population of 210,000 plants ha^−1^ (0.38 m row spacing). The plants were irrigated daily and fertilized continuously until reaching the R3 reproductive stage [24], when the spray tests were conducted. This stage corresponds approximately to BBCH 69, which is characterized by the end of flowering and the onset of pod formation, with pods approximately 5 mm long at one of the four upper nodes on the main stem with fully developed leaves [25].

**Figure 11:**
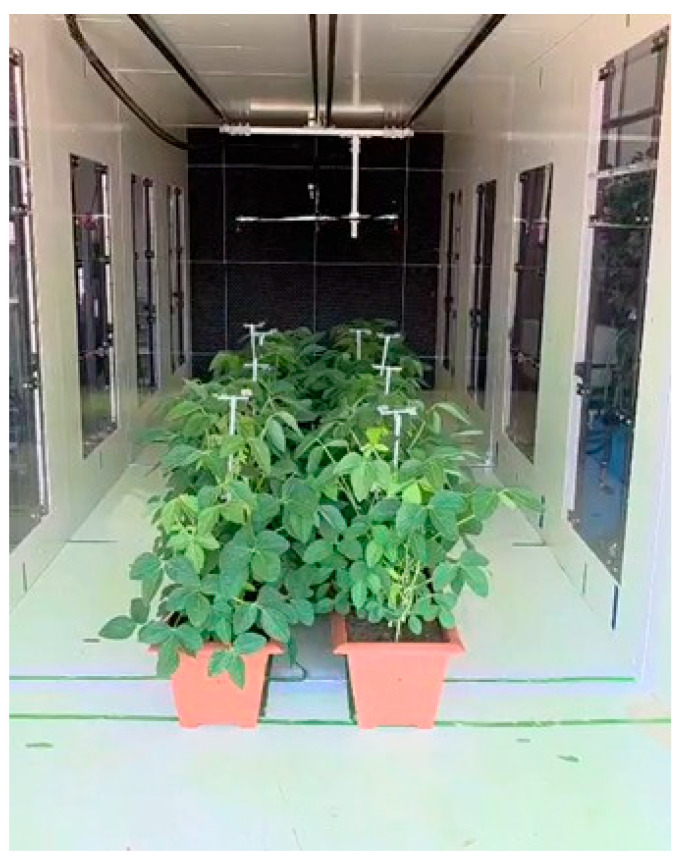
Potted soybean canopy setup in the wind tunnel for LAI and spray deposition measurements.

To replicate field-like growth conditions, the pots were kept outdoors at The Ohio State University Wooster Campus. The Leaf Area Index (LAI) of the potted soybeans was measured using an LAI-2200C Plant Canopy Analyzer (LI-COR Environmental, Lincoln, NE, USA) with two vertically aligned optical sensors, one positioned above and the other below the canopy. Measurements were performed with the pots arranged in the same configuration used inside the wind tunnel and at the eight sampling points of the deposition measurements. Eight soybean pots were arranged in two rows (four pots per row), with a center-to-center row spacing of 0.38 m to reproduce field geometry (Figure 11). Eight readings were taken and averaged across three replications. At the R3 stage, plants had an average height of 0.8 m and LAI of 2.4.

### 3.3. Nozzles

**Figure 12:**
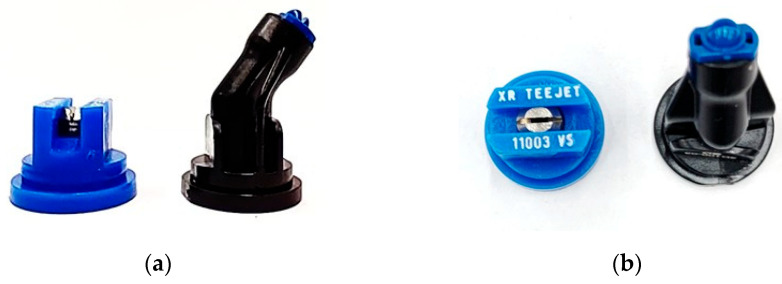
(**a**) Lateral and (**b**) top views of the spray nozzles used in the experiment.

Two types of flat fan spray nozzles (Table 2) were used to evaluate the effect of nozzle type on spray deposition and coverage within the soybean canopy. The XR nozzle produced a standard flat fan spray pattern directed vertically downward. In contrast, the 3D nozzle generated an inclined spray pattern oriented at ±38° relative to the travel direction [26], with the spray fan directed forward or backward depending on the mounting configuration (Figure 12). Each nozzle body was equipped with a 10 Hz pulse width modulation solenoid valve (E-Chemsaver 55295-1-12, TeeJet Technologies, Glendale Heights, IL, USA) to control spray activation. To achieve a spray volume of 130 L ha^−1^ at a travel speed of 0.9 m s^−1^, the nozzle flowrates were modulated to 0.35 L min^−1^ with a 30% duty cycle at the pressure of 276 kPa. The duty cycle was regulated by an EVO spray controller (Capstan Ag Systems Inc., Topeka, KS, USA), which was connected to a smart drive module, boom signal transmitter, and power supply [23].

Droplet size measurements for each spray nozzle were conducted under identical test conditions using a laser imaging particle sizing system (VisiSize N60, Oxford Lasers Ltd., Didcot, UK). Each nozzle was positioned 0.5 m above the measurement area, and approximately 10,000 valid droplets were analyzed for each measurement along the main axis of the spray pattern to characterize the droplet size spectrum. A motorized rail system mounted above the setup was used to laterally move the nozzle at a travel speed of 2 mm s^−1^ across the spray pattern to measure droplet sizes across the entire spray fan.

Once the measurement started, the laser beam from the emitter illuminated the measurement area, and the camera captured droplet images at up to 30 frames per second. The optical system was calibrated for a pixel resolution of 7.6 µm over a 6065 × 4548 µm field of view, allowing accurate measurements of droplet diameters ranging from approximately 20 to 4500 µm.

The following parameters were recorded: D_v10_, D_v50_, and D_v90_ (volumetric diameters at which 10%, 50%, and 90% of the spray volume consists of smaller droplets, respectively); V_153_ (volume percentage of droplets smaller than 153 µm); and the relative span index (a measure of droplet size uniformity, calculated as (D_v90_ − D_v10_)/D_v50_.

### 3.4. Experimental Conditions

Tests were conducted with two air speeds (0 and 4.4 m s^−1^). Soybean plants at the R3 growth stage were arranged in two rows of five pots each (Figure 11), with a 0.38 m center-to-center row spacing, which reflected a common field practice in soybean production. The first pot in each row was positioned 1.88 m downstream from the honeycomb.

To evaluate the influence of wind direction on spray deposition, tests were performed with the spray boom moving both downwind and upwind within the wind tunnel while maintaining the same air speeds (0 and 4.4 m s^−1^). In the downwind treatment (DW), the boom moved in the same direction as the airflow, whereas in the upwind treatment (UW), it moved opposite to the airflow (Table 3). Under these conditions, the relative air speed acting on the spray plume may vary depending on the boom movement direction. In DW conditions, airflow and boom displacement occur in the same direction, resulting in a higher relative travel speed (approximately wind speed + boom speed). In contrast, in UW conditions the airflow and boom displacement occur in opposite directions, leading to a lower relative travel speed (approximately wind speed − boom speed). These differences may influence droplet displacement and spray deposition patterns. Spraying began 1 m before the pots and continued until 1 m beyond them, resulting in a total sprayed distance of approximately 5 m.

Although the practical applicability of the boom travel direction factor under field conditions may be limited, as applicators cannot always control the spraying direction relative to the wind and wind conditions may vary during field operations, this factor was included in the experimental design to evaluate whether the direction of boom movement relative to the airflow should be considered a relevant variable in wind tunnel studies.

### 3.5. Spray Deposition Test

Water-sensitive papers (WSPs) (76 × 26 mm, Syngenta, Basel, Switzerland) were used to quantify spray coverage, while white acrylic plates (APs) of the same dimensions as WSP were employed to assess spray deposition, adapted from the methodology described by Castilho Theodoro et al. [10].

Targets were mounted using twin clip holders attached to eight 1.22-m stakes positioned at the center of soybean pots, corresponding to eight sampling locations in two rows (Figure 11). The stakes supported the targets for subsequent test runs. Each stake had three holders placed at three heights above the soil surface: top (0.80 m), middle (0.40 m), and bottom (0.05 m). The holders were aligned with the wind direction, securing WSPs and APs at three canopy heights across all sampling positions. The collectors were mounted on the stakes rather than directly on the leaves to avoid altering the natural leaf orientation and to maintain consistent positioning across repetitions.

During testing, three nozzles were mounted on the spray boom, and airflow in the wind tunnel was set to the target speed. The 3D nozzle at the center of the boom was oriented to spray forward, while the two lateral 3D nozzles were oriented to spray backward. This configuration remained the same for both upwind and downwind boom movements. This nozzle arrangement is commonly recommended for boom sprayers to increase spray deposition on the lower canopy of crops [26].

For spray deposition measurements, spray solution was deionized water mixed with Brilliant Sulfoflavine (BSF) (MP Biochemicals, Inc., Aurora, OH, USA) at a concentration of 2 g L^−1^.

### 3.6. Sample Assay

After each application, the WSPs were left on the target holders to dry for about 5 min. Then, they were collected and scanned at 600 dpi. Spray coverage (%) was determined by analyzing each WSP using DepositScan [27]. The scanned images were first converted to 8-bit format, and then the software quantified the portion of the WSP surface area covered by spray deposits by calculating the percentage of stained area relative to the total target area.

The APs, once dry, were collected, placed in labeled 110 mL glass jars, and stored in a dark box within a temperature-controlled laboratory. To measure spray deposits (µL cm^−2^) from the APs, deionized water was added to the jars: 40 mL for jars containing APs collected from the top position, and 10 mL for those from the middle and bottom positions. The jars were agitated for 2 min at 200 rpm using an orbital shaker (Solaris 2000, Thermo Fisher Scientific Inc., Waltham, MA, USA) to ensure complete removal of BSF from the APs and full homogenization with the deionized water.

Subsamples (4.5 mL) of the homogenized solution were transferred into polystyrene disposable cuvettes (Fisherbrand, Thermo Fisher Scientific Inc., Pittsburgh, PA, USA) and analyzed using a Trilogy fluorometer (Turner Designs, San Jose, CA, USA) equipped with a custom-made BSF module (BSF 460/500, 7200-4XX) to measure the fluorescence intensity in raw fluorescence units (RFU). A calibration curve was constructed using 15 samples with BSF concentrations ranging from 0.002 to 40.140 mg L^−1^, resulting in a linear regression with a coefficient of determination (R^2^) of 0.999:

BSF concentration (mg L^−1^) = 2.848 × 10^−4^ × RFU − 1.021 × 10^−3^ (1)

BSF concentrations were then converted to spray deposits (µg cm^−2^) based on the wash water volume (10 or 40 mL) and the surface area of each AP (19.76 cm^2^).

### 3.7. Air Velocity and Turbulence

Air velocity and turbulence within the soybean canopy were investigated using a 6-channel constant-temperature anemometry system (StreamLine 9090N0101, Dantec Dynamics, Ramsey, NJ, USA), configured with a three-dimensional (3D) hot-film probe (Type 55, Dantec Dynamics, Ramsey, NJ, USA), to measure airflow along the x, y, and z axes. The probe was calibrated using an automatic 3-D air-velocity calibration unit (StreamLine Pro Calibrator 9090H0101, Dantec Dynamics, Ramsey, NJ, USA) over a range of 0.02 to 40.00 m s^−1^ with errors of less than 0.5%.

Air velocity measurements were taken while the wind tunnel generated wind at a speed of 4.4 m·s^−1^ under the same pot configuration used in the deposition tests, with a 0.38 m row spacing. The probe was mounted on a support to ensure consistent positioning, with the *x*-axis sensor aligned parallel to the airflow direction. Four measurement positions were designated above the soil surface: P1 (above canopy, 1.00 m), P2 (top, 0.80 m), P3 (middle, 0.40 m), and P4 (bottom, 0.05 m). The probes at positions P3 and P4 were placed inside the canopy. Measurements were conducted at the positions used with WSPs and APs during the experiments, targeting the same collection points. Air velocities were measured for 5 s, resulting in 25,000 data points per repetition. Each measurement was repeated three times to ensure accuracy.

As described in detail by Zhu et al. [23], X, Y and Z components of the air velocity (V_x_, V_y_, and V_z_, m s^−1^) in the wind tunnel coordinate system were calculated for each of the 25,000 samples using Equations (2)–(4) [28]:

(2) $$V_{x}=U_{1}cos(54.74{}^{\circ})+U_{2}cos(54.74{}^{\circ})+U_{3}cos(54.74{}^{\circ})$$

(3) $$V_{y}=-U_{1}cos(45{}^{\circ})-U_{2}cos(135{}^{\circ})+U_{3}cos(90{}^{\circ})$$

(4) $$V_{z}=-U_{1}cos(114.09{}^{\circ})-U_{2}cos(114.09{}^{\circ})-U_{3}cos(35.26{}^{\circ})$$

where U_1_, U_2_, and U_3_ are the velocity readings in the local three-wire coordinate system of the 3-D probe.

Subsequently, the resultant air velocity (*V*, m·s^−1^) was calculated with Equation (5):

(5) $$V\;=\;\sqrt{\left(V_{x}^{2}\;+\;V_{y}^{2}+\;V_{z}^{2}\right)}$$

A turbulence intensity (τ, %) of each measurement was computed with Equation (6):

(6) $$\tau \;=\;(\sigma /V_{mean})\;\times \;100$$

where *V_mean_* is the mean resultant air velocity of 25,000 measurements (m·s^−1^), and *σ* is the standard deviation of 25,000-velocity measurements.

### 3.8. Data Analysis

Student’s *t*-test was used to compare droplet spectrum parameters between nozzle treatments. For coverage, deposition, air velocity, and turbulence, the assumptions required for analysis of variance were verified. Normality of residuals was assessed using the Shapiro–Wilk test, and homogeneity of variances was evaluated using Levene’s test. For the data meeting these assumptions, a one-way ANOVA was performed for each sampling position, followed by Tukey’s test when significant effects were detected. The coefficient of variation (CV) for overall coverage and deposition data was calculated for each treatment, aggregating the data from the top, middle, and bottom positions into one data set to verify the uniformity of distribution throughout the plant canopy. Additionally, the Pearson correlation analysis between deposition and coverage was also conducted to examine whether increases in coverage were associated with higher deposition.

All statistical analyses were performed using R software, version 4.2.1 [29], with a significance level of *p* < 0.05 for all tests.

## 4. Conclusions

Wind conditions and boom travel directions had a greater influence on spray coverage and deposition in soybean plants than nozzle types under controlled wind tunnel conditions. These findings emphasized the importance of wind management in pesticide application strategies to ensure optimal spray performance and improve uniformity of canopy penetration.

The top position of the canopy, exposed to the highest air-turbulence intensity out of the top, middle, and bottom positions, received the greatest deposition. However, the middle and bottom positions, characterized by lower turbulence, showed sharp declines in both deposition and coverage regardless of the treatment. This pattern reinforces the substantial challenge of achieving effective spray penetration within the soybean canopy.

Regarding nozzle performance, the 3D nozzle provided better coverage and deposition than the XR nozzle without wind; however, when wind was present, both nozzles performed similarly. Therefore, nozzle choice should be based not only on isolated spray metrics evaluated independently or at a single canopy level, but also on integrated efficacy across the entire canopy under realistic field conditions.

Because this evaluation was limited to a single soybean growth stage, caution should be exercised when extrapolating these findings to other developmental phases. Future research should encompass multiple growth stages and prioritize the development of predictive models that incorporate droplet size spectrum, wind conditions, and canopy structures to optimize application parameters for efficient and effective crop protection.

## Figures and Tables

**Table 1:** Volumetric droplet diameters (D_v10_, D_v50_, D_v90_), V_153_, and relative span index of sprays discharged from XR 11003 and 3D 10003 nozzles operated at 30% duty cycle and 276 kPa pressure.

Nozzle	D_v10_ (µm)	D_v50_ (µm)	D_v90_ (µm)	V_153_ (%)	Relative Span Index	Droplet Size Classification ^1^
XR 11003	94 b	164 b	367 b	44.8 a	1.6 a	Fine
3D 10003	118 a	248 a	538 a	23.0 b	1.7 a	Medium

Means followed by different letters within a column are significantly different according to Student’s *t*-test (*p* < 0.05). ^1^ According to ANSI/ASABE S572.3 standard [11] at the evaluated conditions.

**Table 2:** Characteristics of the nozzles used in the experiment.

Nozzle Type	Model	Nominal Size	Spray Angle (Degree)	Droplet Size Classification ^1^	Pressure (kPa)
Extended range flat fan	XR ^2^	03	110	Fine	276
Alternating flat fan	3D ^3^	03	100	Medium	276

^1^ According to the manufacturer. ^2^ Nozzle was from Spraying Systems Co. (Glendale Heights, IL, USA) ^3^ Nozzle was from Pentair (Golden Valley, MN, USA).

**Table 3:** Description of experimental treatments based on nozzle type, spray boom direction, and air speed.

Treatment	Nozzle	Spray Boom Direction	Air Speed (m s^−1^)
3D-UW-0.0	3D	Upwind (UW)	0.0
XR-UW-0.0	XR	Upwind (UW)	0.0
3D-DW-0.0	3D	Downwind (DW)	0.0
XR-DW-0.0	XR	Downwind (DW)	0.0
3D-UW-4.4	3D	Upwind (UW)	4.4
XR-UW-4.4	XR	Upwind (UW)	4.4
3D-DW-4.4	3D	Downwind (DW)	4.4
XR-DW-4.4	XR	Downwind (DW)	4.4

## Data Availability

The original contributions presented in this study are included in the article. Further inquiries can be directed to the corresponding author.
