# Correction: Cunha et al. Soybean Foliar Deposition and Airflow Distribution Interrelated to Nozzle Type and Boom Travel Direction in Wind Tunnel. *Plants* 2026, *15*, 1032

**DOI:** 10.3390/plants15091413

**Published:** 2026-05-06

**Authors:** João Paulo Arantes Rodrigues da Cunha, Rone Batista de Oliveira, Gabriel de Souza Lemes, Erdal Ozkan, Hongyoung Jeon, Heping Zhu

**Affiliations:** 1 Institute of Agrarian Sciences, Federal University of Uberlândia, Uberlândia 38408-100, Brazil; 2 Department of Food, Agricultural and Biological Engineering, The Ohio State University, Wooster, OH 44691, USA; rone@uenp.edu.br (R.B.d.O.); desouzalemes.1@osu.edu (G.d.S.L.); 3 Center of Agrarian Sciences, Northern Paraná State University, Bandeirantes 86400-000, Brazil; 4 Department of Food, Agricultural and Biological Engineering, The Ohio State University, Columbus, OH 43210, USA; ozkan.2@osu.edu; 5 Application Technology Research Unit, United States Department of Agriculture, Wooster, OH 44691, USA; hongyoung.jeon@usda.gov (H.J.); heping.zhu@usda.gov (H.Z.)

**Text Correction**

There was an error in the original publication [1] regarding the description of plant arrangement in Section 3.2. *Soybean Plants*.

A correction has been made to Section 3.2:

“Each pot contained eight plants arranged in a single central row, corresponding to a field population of 310,000 plants ha^−1^ (0.38 m row spacing).”

“Ten soybean pots were arranged in two rows (five pots per row), with a center-to-center row spacing of 0.38 m to reproduce field geometry (Figure 11).”

The authors state that the scientific conclusions are unaffected. This correction was approved by the Academic Editor. The original publication has also been updated.
